# Health workers’ perspectives, knowledge and skills regarding community case management of childhood diarrhoea and pneumonia: a qualitative inquiry for an implementation research project “Nigraan” in District Badin, Sindh, Pakistan

**DOI:** 10.1186/s12913-016-1699-5

**Published:** 2016-09-01

**Authors:** Fauziah Rabbani, Shagufta Perveen, Wafa Aftab, Aysha Zahidie, Kashif Sangrasi, Shamim Ahmad Qazi

**Affiliations:** 1 Department of Community Health Sciences, The Aga Khan University, Stadium Road, P.O. Box 3500, Karachi, 74800 Pakistan; 2 Department of Maternal, Newborn, Child and Adolescent Health, World Health Organization, Geneva, Switzerland

**Keywords:** Implementation research, Community health workers, Integrated community case management, Childhood diarrhoea and pneumonia, Qualitative inquiry, Pakistan

## Abstract

**Background:**

Pakistan’s Lady Health Worker Programme aims to provide care to children sick with pneumonia and diarrhoea, which continues to cause 27 % under-five mortality in Pakistan. The quality of supervision received by Lady Health Workers (LHWs) in the programme influence their knowledge and skills, in turn impacting their ability to provide care.

**Methods:**

This study is part of an implementation research project titled “Nigraan” (an Urdu word meaning supervisor), and explores LHW and Lady Health Supervisor (LHS) perspectives regarding the role of supervision in improving LHWs performance and motivation in district Badin, Sindh, Pakistan. Their knowledge and skills regarding integrated community case management (iCCM) of diarrhoea and pneumonia were also assessed. Fourteen focus group discussions and 20 in-depth interviews were conducted as part of this qualitative inquiry. Analysis was done using QSR NVivo version 10.

**Results:**

Most LHWs and LHSs identified pneumonia and diarrhoea as two major causes of death among children under-five. Poverty, illiteracy, poor hygiene and lack of clean drinking water were mentioned as underlying causes of high mortality due to diarrhoea and pneumonia. LHWs and LHSs gaps in knowledge included classification of dehydration, correctly preparing ORS and prescribing correct antibiotics in pneumonia. Lack of training, delayed salaries and insufficient medicines and other supplies were identified as major factors impeding appropriate knowledge and skill development for iCCM of childhood diarrhoea and pneumonia. LHWs considered adequate supervision and the presence of LHSs during household visits as a factor facilitating their performance. LHWs did not have a preference for written or verbal feedback, but LHSs considered written individual feedback to LHWs to be more useful than group and verbal feedback.

**Conclusion:**

LHWs have knowledge and skill gaps that prevent them from providing effective care for diarrhoea and pneumonia. Enhanced supportive feedback from LHSs could improve LHWs skills and performance.

## Background

Pneumonia and diarrhoea are responsible for around 1.5 million deaths per year in children under five globally [1]. In Pakistan, pneumonia and diarrhoea contribute to 27 % of all deaths in children under five [2]. Furthermore, approximately 41 % of children under five receive the recommended antibiotics for suspected pneumonia and oral rehydration therapy for diarrhoea [3].

Community health workers (CHWs) can play an important role addressing pneumonia and diarrhoea as primary community caregivers providing the first line of care in the health system [4]. CHWs are geographically closer and more readily available than health facilities, and as residents of the local community they can provide care without cultural and linguistic barriers. Therefore their knowledge and skills are vital in providing integrated community case management (iCCM) of pneumonia and diarrhoea. In rural settings of Afghanistan, Bangladesh, Iran, Indonesia, Brazil and Nepal, CHW promotion of household and community health practices is an important strategy to improving child health [5, 6]. For example, CHWs in India, Bangladesh and Nepal trained to assess, classify and treat pneumonia were capable of managing pneumonia adequately in the community [7]. The BRAC CHWs known as Shasthya Shebika (health sisters) have made considerable contribution to Bangladesh’s progress in reducing under five mortality [6]. The community initiative for child survival project in Siaya District has improved child survival by training and supporting Kenyan CHWs [8]. Ethiopia has also involved CHWs extensively in delivering iCCM [9]. In a study in Uganda, CHWs had difficulty linking diagnostic results to classification and only 40 % of children with pneumonia were prescribed an antibiotic [10]. Incorrect diagnosis was a key problem which preceded two-thirds of all treatment errors. In each of these studies, CHWs had deficiencies in clinical skills and faced several logistic obstacles in providing care to children with pneumonia and diarrhoea [8, 9].

In Mali gaps were identified in the knowledge and practices related to home management of diarrhoea by CHWs [11]. The study reported that training, motivation, supervision and continued mentoring in the field were important considerations for poor performance of CHWs and must be appropriately addressed [11]. Gopalan has categorized CHW motivation into individual, community and health system levels. Understanding these factors is important to the efficiency of healthcare delivery [12].

In Pakistan, the Lady Health Workers Programme (LHW-P) was launched in 1994 to bring health services closer to the communities. One of the objectives of the programme was to provide care for children with pneumonia and diarrhoea. Currently it covers 60 % of Pakistan’s rural population and there are approximately 1, 30,000 Lady Health Workers (LHWs) deployed throughout Pakistan.

As primary community care givers, LHWs address uptake of maternal and child health services, such as family planning and antenatal care, in rural areas of Pakistan. However, they have had minimal impact on increasing coverage of pneumonia and diarrhoea treatment [13]. The Fourth External Evaluation of the National Programme (FENP) identified weak LHW knowledge and skills due to insufficient supervision by Lady Health Supervisors (LHSs) as a reason for this problem [13].

There is a growing body of qualitative research into LHWs training needs and the social and economic impact of the LHW–P on health indicators in Pakistan [4, 14]. However, these studies do not explore health worker perspectives, knowledge and skills regarding iCCM of childhood diarrhoea and pneumonia, or the role of supervisory approaches in improving LHWs’ performance. This implementation research is part of “Nigraan” (an Urdu word meaning supervisor), a randomized controlled trial (RCT) based in rural Pakistan designed to improve iCCM of childhood diarrhoea and pneumonia by LHWs and LHSs [15]. The purpose of this study is to explore LHWs and LHSs perspectives on the role of supervision in improving LHWs performance and motivation and their knowledge and skills for iCCM of diarrhoea and pneumonia.

## Methods

An exploratory qualitative study using focus group discussions (FGDs) and in-depth interviews (IDIs) with LHSs and LHWs was conducted in district Badin, Sindh, Pakistan.

### Site characteristics

District Badin is an area of 6726 square kilometers divided into five Talukas and 46 Union Councils. The total population is 11,93,081, of which 81 % are Muslim and approximately 17 % are Hindus [16]. Muslim beliefs and culture predominantly influence care seeking and decision making at the household level in Badin. Table 1 summarizes the infrastructure of health facilities in Badin [17].

**Table 1:** Number of health facilities in Badin

Health facility	Number
District Headquarter Hospital	01
Taluka Hospitals	04
Rural Health Centres	12
Basic Health Units	35
Dispensaries	15
Maternity Homes	03

*Source:* Disaster risk management plan: district Badin Government of Sindh - 2008

### LHW programme and district administrative structure

In Pakistan, LHWs are a major outreach component of the primary health care system, as illustrated in Table 2. There are a total of 1105 LHWs in Badin supervised by 36 LHSs that cover 55 % of the area [18]. Each LHS represented a cluster in the Nigraan RCT. Under the programme, an LHW is the point of care for the community. She provides preventive and basic curative maternal, newborn and under five child health (MNCH) services in her catchment area. LHWs are salaried staff, preferably married and educated (minimum eight years of schooling) and mostly reside in the area where they serve. An LHW serves approximately 100–150 households, representing an average population of 1000 [12]. The LHW also works from her home, where she is encouraged to have a portion of her home designated as a “Health House” [19].

**Table 2:** Province wise coverage of LHW Programme in Pakistan

S. No.	Province	Number of working LHWs	Number of Supervisors working	% population covered
1.	Sindh	22767	782	46 %
2.	Punjab	49887	1808	55 %
3.	KPK	13702	470	58 %
4.	Baluchistan	6674	241	28 %
	Total	93030	3301	

*Source:* Reports on the status of MDGs - all provinces – UNDP and Provincial Government (Pakistan). 2008

LHSs are attached to the first level care facility (FLCF) and are responsible for on-going supervision and monitoring of LHWs according to a predefined standard checklist. LHSs are female health workers aged 22–45, residing locally with a good educational background and have several years’ experience as a Lady Health Visitor (LHV) or LHW. LHS salaries range from 160 to 180 USD per month. Each LHS supervises approximately 15–25 LHWs and is supposed to visit one LHW twice a month, accompany them during their home visits, guide them and address their concerns. LHWs report to LHSs and submit their progress reports once per month. LHSs in turn report to the Assistant District Coordinator (ADC) based at the District Program Implementation Unit (DPIU).

LHSs use supportive supervision, which is part of the national LHS curriculum. It is defined as “a supervision strategy in which every supervisor knows about the needs of his/her subordinates. The supervisor tries to improve the performance of staff to achieve the goals of the programme. In this strategy good communication and mutual problem solving is highly emphasized between a supervisor and staff. The supervisor is considered a bridge between staff and administration” [20].

### Study participants

Nigraan RCT included 34 functional LHSs and five LHWs working under each LHS (total 170). Two LHSs were excluded because one was reported to be non-functional and the other was already serving as ADC Badin. Out of 34 included LHSs one LHS could not participate due to personal reasons.

Two LHWs were selected randomly from each cluster to participate in FGDs. One LHW was selected from every fourth cluster to participate in IDIs. The project field team built rapport with study participants before commencing each FGD and IDI, and the ADC Badin assisted in coordinating the participation of these LHWs. Fourteen FGDs and 20 IDIs were conducted, as detailed in Table 3.

**Table 3:** Total number of participants in FGDs and IDIs

Participants	FGDs	IDIs	Total
LHS	23	10	33
LHWs	65	10	75
Total	88	20	108

### Data collection

Data collection involved FGDs and IDIs. A semi-structured guide (Table 4) with pre-determined list of open-ended questions, arranged in a logical sequence was used in all FGDs and IDIs. Author SP designed this guide with the research team. This guide was translated into the local language (Sindhi) and then back translated into English. The pilot testing was done in two similar settings which resulted in precision of some questions and addition of specific probes especially under the knowledge and skills section. The final guide included questions on LHWs/LHSs knowledge and skills regarding pneumonia and diarrhoea, motivation, supervisory process, training and feedback mechanisms.

**Table 4:** Probes in FGD and IDI guides

S. No.	Sections	Main Probes
1.	Opening questions	• Perception about most important causes of death among children under five • Opinion about LHW Programme’s capacity to address these causes • Perception of primary caregivers about the role of LHWs in the community
2.	LHWs/LHSs knowledge and skills regarding diarrhoea	• Definition, classification, diagnosis, treatment. • Barriers and facilitators for enhancing health workers’ learning and training
3.	LHWs/LHSs knowledge and skills regarding pneumonia	• Definition, classification, diagnosis, treatment. • Barriers and facilitators for enhancing health workers’ learning and training
4.	LHWs/LHSs Motivation	• Motivating and demotivating factors influencing job performance (training, salary, supplies etc.)
5.	Supervisory process	• Importance and effect of supervision on performance • Required competencies for optimum supervision • Satisfaction level and suggestions for improving current supervision
6.	Feedback	• Existing mechanisms of feedback to LHWs from LHSs • Suggestions for improving feedback

All FGDs and IDIs were conducted in Sindhi language at the LHW–P office, Badin in 2014. Author SP was present in all FGDs and IDIs assisted by an experienced moderator. All FGDs and IDIs were audio-recorded using a digital recorder. A separate note taker was also present in all FGDs and IDIs making field notes. Each FGD lasted for around 70–80 min and each IDI for about 40–45 min.

### Data analysis

All FGDs and IDIs were transcribed in English and all the transcripts were read through and brief notes were made at the points of interesting and relevant information. The data analysis process was inspired by Braun and Clark’s thematic analysis method. Researchers began by familiarizing with the data, generating codes, searching, reviewing and naming themes [21]. Relevant probes were picked from the transcribed data using the software QSR NViVo version 10 and linked to the identified theme. All disagreements were discussed and consensus reached on the overall analysis. Several common themes emerged from categorization. All the transcripts were reviewed again in the end to make sure that the necessary information had been captured. The researchers shared preliminary findings with study participants, whose feedback was then integrated into the final stage of the data analysis process.

## Results

The age of participants ranged from 25 to 56 years with a minimum 8 years of schooling. LHWs had worked with the LHW-P between 5 and 10 years, and LHSs between 5 and 22 years.

### Pneumonia and diarrhoea: the major killers

LHWs and LHSs perceived pneumonia and diarrhoea as the two top causes of under-five child mortality, along with measles, malnutrition and anemia.

The responses from LHWs and LHSs identified several factors at the household or facility level that contribute to pneumonia and diarrhoea mortality. These include, use of traditional remedies, lack of proper care and treatment, and reducing the child’s food intake.

“*People stop feeding the child during diarrhoea*” *FGDLHW05*

Limited access to LHWs in some areas was also mentioned as a reason for compromised care at household level.

“*LHWs can’t reach few places; a great need is there*” *FGDLHW02*

“*People can’t reach the hospital and use traditional remedies at home instead*”. *IDILHW01*

LHWs and LHSs were aware that immunization status is linked to risk of pneumonia. They believed that inappropriate vaccination status, inadequate caregiver knowledge and malnutrition contribute to childhood mortality. Unhygienic practices, unsafe drinking water and bottle feeding were also mentioned.

### LHWs and LHSs knowledge and skills for pneumonia and diarrhoea

#### Diarrhoea

Almost all LHWs and LHSs were able to define diarrhoea correctly as three or more loose watery motions in 24 h. However, LHWs and LHSs did not have in-depth knowledge of classification and typology of diarrhoea such as acute, chronic and dysentery. When asked about how they assessed and managed a child with diarrhoea in community, the most common response was giving ORS followed by referral. None of the participants mentioned assessment of dehydration as part of diarrhoea case management plan.

When LHWs and LHSs were specifically probed about assessing for dehydration, the majority correctly mentioned to check the skin and eyes. However, no one pointed out consciousness, eagerness to drink water and ability to feed as other features of dehydration. Classification of dehydration into mild, moderate and severe was also not described.

Consequently, when talking about the management of diarrhoea most LHWs mentioned giving ORS but no one related ORS administration to the degree of dehydration or weight of the child, as per information given in LHW–P curriculum. Zinc supplementation was not mentioned by any of the participants as part of diarrhoea treatment. This could be due to the inadequate supplies of zinc available with the LHW–P.

Similar findings emerged from FGDs and IDIs conducted with LHSs. However, LHSs were able to comment more comprehensively on the types of diarrhoea and method for assessing dehydration.

“*There are categories: the child is drinking, drinking with difficulty and not drinking at all” FGDLHS02*

#### Pneumonia

Most LHWs and LHSs were able to correctly define pneumonia as chest indrawing, high grade fever and fast breathing. However, few misconceptions were noted among LHSs with regard to pneumonia.

*“Pneumonia is inflammation of hands” FGDLHS01*

*“Inflammation of intestine is pneumonia” FGDLHS02*

*“Pneumonia is ear, nose and throat problem” FGDLHS01*

LHWs were not able to classify or identify pneumonia, whereas LHSs were more confident in their ability to identify pneumonia. Upon identification of pneumonia, LHWs and LHSs tend to refer the child to the next level of care. Very few LHSs or LHWs mentioned conducting a disease assessment, but both LHWs and LHSs mentioned that they counsel caregivers to keep the child warm and prescribe cotrimoxazole.

Books and charts provided by the LHW–P were considered important to acquiring and maintaining knowledge about pneumonia and diarrhoea. However, some of the workers mentioned that they did not have the charts with them anymore.

“*We don’t have charts now, destroyed in rains, new cards should be available” FGDLHW02*

While training was understandably mentioned as important to improve level of knowledge, availability of medicines was considered equally important.

*“Our level of knowledge will increase if we get medicines” FGDLHW02*

**Fig. 1:**
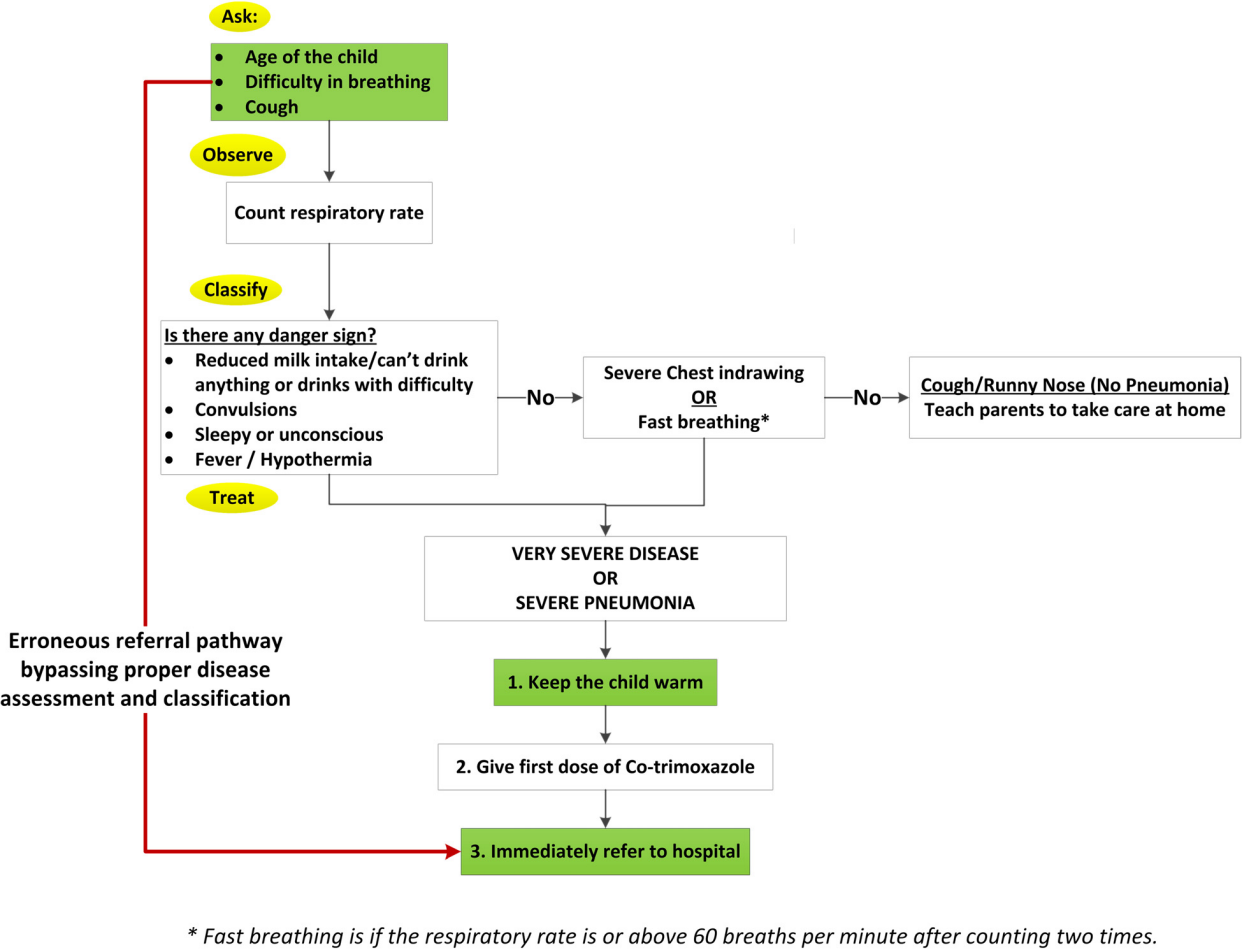
LHW Programme management protocol for acute respiratory infection - Gaps in LHW compliance standards

Figure 1 depicts the standard protocol for the management of acute respiratory infections in the LHW–P. We have used this template to demonstrate the steps which are required but not practiced by the health workers while visiting a sick child (un-shaded boxes). The steps which are actually performed at the household level are shown in shaded boxes.

### Salary, medicines and supplies: major motivators

LHWs and LHSs reported financial need as the major reason for joining LHW–P, followed by service to the community and interest in the health profession.

The motivation of all LHWs and LHSs is significantly hampered by the lack of supplies and medicines. A considerable number of health workers highlighted the unavailability of ORS, zinc and antibiotics as a demotivating factor in terms of improving knowledge and performance. For instance, health workers have not received any antibiotic to treat pneumonia for five years. Health workers believe that the availability of medicines can strengthen their credibility in the community.

*(People in the community say) “you don’t give us medicines; you pocket them yourselves”. FGDLHW05*

Recognition of their work and appreciation by the supervisor has an encouraging effect on LHWs’ performance.

“*If we get these four things then we can perform our duties easily; Salary, material, training, reward” FGDLHS02*

### Recognition of supervision as an essential element of work

While none of the health workers had heard about the concept of “supportive supervision” almost all LHWs mentioned the characteristics corresponding to the idea.

“*a good supervisor should be responsible, friendly, well-mannered, soft-spoken, well-informed, experienced, doing regular field visits and respectful of others”. FGDLHW03*

LHWs and LHSs were of the opinion that enhanced presence of LHSs during their household visits would be a source of encouragement and improved LHW performance.

This is because LHWs think that LHSs are fully trained for diarrhoea and pneumonia so supervision corrects their mistakes, provides guidance and boosts their confidence. According to many LHWs the supervision would be significantly improved if the LHSs accompany them on field visits more often.

*“Its better to go and check (LHWs) in the field then to ask only.” IDILHW04*

However, LHSs pointed out that lack of transport and fuel is a major factor preventing them from making more frequent field visits and on site supervision.

### Feedback process improvement

LHWs and LHSs perceived feedback as being important to improving their performance.

*“Feedback is very important, in this way our capabilities are exposed.” FGDLHW09*

LHWs get group feedback on their overall performance during monthly meetings. Generally LHWs preferred group feedback as compared to individual feedback. The latter could be due to fear of reprisal. There was no clear LHW preference for either written or verbal feedback from their LHSs. Most LHWs consider verbal feedback given in a group to be adequate. In some instances, LHWs actually prefer verbal over written feedback as they can comprehend and retain verbal messages better than written ones. Written feedback is perceived with anxiety by LHWs because it becomes a documentary proof of performance labeling someone “good” or “bad” without actually observing them in the field.

*“Individual feedback is useless; no one knows what we did” FGDLHW06*

*“It will be beneficial to give feedback in a group” FGDLHW09*

On the other hand, LHSs preferred written and individual feedback to LHWs, rather than group and verbal feedback. They think that positive feedback should be given in the group and negative individually. Written feedback should be used as reinforcement in case verbal messages do not improve performance.

*“Existing group feedback mechanism can be improved by giving feedback in written form” FGDLHS04*

*“Written feedback is more important. It always remains with LHWs and is more effective” FGDLHS02*

*“Feedback should be written and given individually” LHSIDI06*

### Training

The need for regular training was repeatedly emphasized by participants. According to health workers further training would improve LHWs’ knowledge, performance, motivation and LHSs’ supervision. Almost all of the LHWs and LHSs in this study indicated that the supervisors must be trained further to improve their knowledge and skills to clinically mentor LHWs.

The last training for most LHWs and LHSs was conducted several years ago, but they desired additional observation-based training to enhance their knowledge and skills.

Training was also stated by all LHSs and LHWs as one of the major motivating factor to perform better. It was also perceived that training would improve iCCM of diarrhoea and pneumonia.

*“More training should be provided to improve knowledge regarding diarrhoea and pneumonia” LHSIDI05*

A strong desire to gain more knowledge was also expressed especially in the absence of formal training for several years.

*“Training is very important, if we forget anything training helps to recall and stimulates our memories” FGDLHW07*

*“The more increase in knowledge, the better it is. It is definitely possible that there are things which we don’t know” LHSIDI08*

### LHWs perceived as “Polio Workers” by the community

Many participants reported that the community recognizes LHWs as “vaccinators” because they visit households for polio campaigns. Therefore, LHWs have become more recognized for their polio work than their designated LHW-P responsibilities. Some community caregivers inquire with the LHWs about the next vaccination campaigns rather than for other MNCH issues.

“*Polio person has come*” *FGDLHW06*

*“In the beginning, the people did not like us and our work but now they contact us themselves as they need treatment and advice especially during polio campaign” FGDLHW02*

*“Yes, definitely, initially people were unaware and did not allow for polio vaccination, but now they call us to vaccinate their children” IDILHW02*

## Discussion

The two most important causes of deaths among children under five as perceived by health workers were pneumonia and diarrhoea along with measles, malnutrition and anaemia. This shows that health workers have appropriate knowledge regarding underlying factors contributing towards child morbidity and mortality and their perception coincides with the global and national trends of under-five mortality [22–24].

LHW and LHS in-depth knowledge about the management of diarrhoea and pneumonia was limited in our study. This could be due to the lack of regular training, irregular supply of medicines and inadequate application of practical skills in iCCM. As a consequence, their first response tends to be referral of the sick child to the nearest health facility without assessing and classifying the disease. Therefore, despite the presence of a rich primary health care infrastructure the functionality and quality of care provided by these health workers is a major issue. Hence community care providers resort to seeking care at less accessible tertiary care hospitals.

Our findings on health worker’s deficient knowledge to identify and classify pneumonia correspond with a WHO survey in 57 African and Asian countries which indicates that CHWs had difficulty assessing pneumonia in children. In particular, there is a need for training them in the identification and management of childhood pneumonia and in clinical skills like counting respiratory rate, and looking for chest in-drawing [25]. There is a strong need for providing skills-oriented regular training to health workers in order to improve iCCM of diarrhoea and pneumonia.

With regard to factors which tend to demotivate health workers from giving optimum performance; lack of salary, training and medicines demotivate LHWs and LHSs. This matches well with Franco et al’s conceptual framework to explain interaction between various motivational determinants and processes for health workers [26, 27]. It may be noted that the LHW-P in Pakistan has only recently installed a regular salary structure. This combined with uncertainty about job status has led to demotivation amongst health workers who work amidst difficult circumstances in rural areas.

In a society that is highly curative oriented [28], the inability to provide medicines reduces the credibility of LHWs and LHSs in the community. It is therefore understandable that these workers are best acclaimed as “polio workers” because at that time they are positively viewed as providers of polio vaccines. Polio coverage in the province is 70 % [29]. LHWs go for polio vaccination regularly [30]. The downside of this is that LHWs routine responsibilities related to their larger MNCH role are compromised because the community does not recognize them for their actual mandate.

Similar findings have been reported from other studies in rural Tanzania and Rwanda [31, 32]. In North Vietnam, salaries and poor working conditions discourage public health workers in rural areas to perform better [33]. Another study in Mali documents that salary and recognition for work motivates health workers to perform better. The same study pointed out that lack of materials and equipment are demotivating factors for health worker related job responsibilities [34]. In a review by Rowe et al., lack of supervisory skills and transportation emerged as the key challenges for supervising CHWs in Zimbabwe and other low resource settings [35].

Most LHWs in this study perceived supportive supervision by their LHSs as being a mediator towards better performance. However they clearly pointed out that benefits are more if the system ensures that it is not carried out as a punitive process. Other studies in low middle income countries also report the need for training or re-training supervisors as an effective way to bring improvement in the supervisory process [14, 36, 37].

None of the health workers in this study were aware about supportive supervision as a concept. This is not surprising because even though it is part of the LHW-P curriculum, the concept and attributes of supportive supervision are not part of their training. These weak LHSs supervisory and clinical mentoring skills translate into poor LHWs case management [38]. Therefore, this study illustrates the importance of making supervision and mentorship of LHWs a regular part of LHSs’ practical training.

Feedback was considered by LHWs as an important factor to improve their performance but there was no clear consensus regarding the method of providing feedback (written vs. verbal and group vs. individual). The literature also indicates that feedback has significant contribution in improving health workers’ performance [35]. One of the reasons that health workers preferred group over individual feedback could be that to date they have not been provided quality feedback on a one to one basis. However, the LHSs indicated the usefulness of both group and written feedback as significant means to improve their performance.

All health workers considered regular training the best measure to improve their work. Studies from Pakistan and Mali indicate that continuous training, availability of transport, adequate supervision and motivation of CHWs through regular remuneration and appreciation are among key factors to improve their performance in rural communities [11, 28, 39, 40]. Hence it is encouraged that evidence-based packages be used for training LHWs to enhance their skills for iCCM of pneumonia and diarrhoea [41].

In terms of methodological considerations, trustworthiness remains a challenge in qualitative research. We have tried to ensure representation from all districts in the sample by suing multiple sources of information to add credibility, selecting participants randomly for FGDs and IDIs and conducting frequent debriefing sessions with the study participants. In order to address transferability the profile of study site and the LHW-P has been provided in detail so that phenomenon under investigation can be contextually interpreted.

## Conclusions

Recognition, supportive supervision, training, logistics and salaries emerged as factors that have the potential to improve health workers’ motivation for better application of their knowledge and skills. These factors can be explored further in other CHW programs in the region to improve CHW performance. They are all directly influenced by LHW-P policies and should be the focus for resource allocation at the program level. Moreover these system level determinants are integral to health worker’s motivation. Hence these motivational factors need to be further evaluated in all health system interventions embedded in implementation research projects.

## Abbreviations

ADC: Additional district coordinator
CHWs: Community health workers
DPIU: District programme implementation unit
ERC: Ethical review committee
FGDs: Focus group discussions
FLCF: First level care facility
iCCM: Integrated community case management
IDIs: In-depth interviews
LHSs: Lady health supervisors
LHW-P: Lady health worker programme
LHWs: Lady health workers
WHO: World health organization
